# Randomized clinical study to compare implant stability and bone loss between different implant designs

**DOI:** 10.6026/973206300220180

**Published:** 2026-01-31

**Authors:** Anchal Bhardwaj, Koguru Madhavi, Prashant Patil, Asad Mujawar, Sweta Singh, Manaswini Draksharapu, Md Kafeel Ahmed

**Affiliations:** 1 Department of Dentistry, ESIC Medical College & Hospital, Alwar, Rajasthan, India; 2 Department of Periodontology and Implantology, Consultant, Shine n Smile advanced dental specialty, Hyderabad, Telangana, India; 3 Department of Orthodontics, Al Badar Dental College and Hospital, Gulbarga, Karnataka, India; 4 Department of Prosthodontics and Implantology, Private Practitioner, Mazaya Dental Center, Muhayil, Saudi Arabia; 5 Department of Oral and Maxillofacial Surgery, ESIC Dental College and Hospital, New Delhi, India; 6 Department of Prosthodontics Crown Bridge and Implantology, Mamata Dental College, Khammam, Telangana, India; 7 Department of Periodontology and Implantology, MNR Dental College and Hospital, Sangareddy, Hyderabad, Telangana, India

**Keywords:** Dental implant design, implant stability, marginal bone loss, tapered implant, resonance frequency analysis, split-mouth study

## Abstract

Competing macro geometric designs of implants allegedly determine the primary stability and long-term marginal bone maintenance though the sound of randomized evidence is hazy. This split-mouth randomized controlled trial was a comparative study of the tapered and straight design of implants in terms of stability and crestal bone loss in 12 months. Sixty bilateral missing first mandibular molars patients were given one type of implant on a side (tapered, progressive-thread; straight, standard-thread), randomly distributed. Resonance frequency analysis at placement, 3, 6 and 12 months was used to measure implant stability quotient, marginal bone loss was measured at 12 months through cone-beam computed tomography. Tapered implants were found to have much higher mean ISQ at placement (72.4±4.8 vs 68.9±5.2, p=0.001) and at three months (75.1±4.2 vs 72.3±4.9, p=0.007) and thereafter. The loss of crestal bone in tapered implants was 0.82+0.31mm compared to the straight implants that had 1.34+0.45mm (p<0.001). The tapered design of the implants helps to provide initial stability and reduce crestal bone resorption and this is in support of its application in the posterior mandibular position.

## Background:

The field of dental implant therapy has developed an alternative form of predictable modality in the rehabilitation of edentulous spaces and has been reported to have a survival rate of above 95% in a period of 10 years [1]. Although the results of this are positive, peri-implant marginal bone loss is a significant parameter that determines the success in the long term and predictable aesthetics [2]. The mean loss is 1.0 -1.5 mm bone loss in the first year and an annual bone loss of 0.1- 0.2 mm is deemed as acceptable [3]. Unregulated bone resorption endangers the support of soft tissue, impairs the harmony of the implants and the prosthetic and heightens the risk of peri- implantitis [4]. In turn, defining the modifiable factors that mitigate bone loss presupposes the primary clinical significance. The macroparticle of the implant is also one of the key determinants of the initial mechanical stability as well as the future biological reaction. There are two major design options, which include straight-walled implants with standard threads and tapered implants with progressive thread geometry. Tapered designs supposedly improve primary stability by compressing the apical bone which is beneficial especially in extraction sockets or lower bone densities [5]. In addition, the progressive thread pitch can be beneficial to optimize the load transfer and reduce the micromotion and thereby reduce the strain in the crestal bone [6]. These theoretical benefits are supported with finite element analysis which show that 15-20 percent lower stress concentrations at the crestal are actually achieved when using tapered designs as compared to straight designs [7]. Nevertheless, these biomechanical predictions have not been validated in the clinical sense. A randomized trial comparing tapered and straight implants in the posterior maxilla (2018) showed that there was no significant difference between the two types in terms of stability at three months but bone loss was reduced with tapered- designed implants by 0.4 mm [8]. On the other hand, a prospective cohort study reported excellent primary stability of straight implants in dense mandibular bone, which was related to the increased area of contact with the surface [9]. These conflicting results would presumably be due to the methodological heterogeneity, such as dissimilar measurement protocols, variable loading protocols and insufficient control of confounding factors, such as bone density and surgical technique. In addition, the majority of studies adopt parallel-group designs, which add inter-individual variation, which can confound small effects driven by design.

Objective, reproducible stability assessment can be determined through resonance frequency analysis (RFA) by use of implant stability quotient (ISQ) and correlates with micromobility and histomorphometric bone-to-implant contact [10]. Whereas primary stability is a result of mechanical involvement, secondary stability is a result of the process of osseointegration, which is normally reflected through the increase of ISQ 36 months [11]. At the same time, the marginal bone level assessment through the standardized radiography or cone-beam computed tomography (CBCT) measures the responses to peri-implant remodelling [12]. The alignment of these measures in the strict randomized system can provide a complete understanding of the implant performance patterns. The split-mouth design is the methodological ideal of comparing implant systems because it removes the confounding variables, such as patient-specific variables like bone metabolism, oral hygiene and loading patterns [13]. Astronomically, very little split-mouth tests have been done to determine influences of macro design, most of the studies are based on surface modifications or types of connections [14]. Such deficiency in good evidence does not facilitate evidence-based selection criteria of implant design in particular clinical situations. Therefore, it is of interest to compare the stability of implants and code of marginal bone loss with tapered and straight designs in the anterior segment of the mandible in a split-mouth model.

## Materials and Methods:

## Study design and setting:

It was a prospective, examiner-blind, split-mouth randomized controlled trial that was carried out at the Department of Oral and Maxillofacial Surgery, between February, 2022 and March, 2024.

## Sample size calculation:

The estimation of sample size was made on the pilot study of 12 patients which revealed a difference of 0.5 mm between designs in marginal bone loss (SD 0.6 mm). A paired t-test with 0.05 40 which has a power of 0.80 and an effect size of 0.83 would have required a minimum of 26 pairs. The number enrolled patients were anticipated to be 15% dropout: 60 patients (120 implants).

## Participant selection:

Sixty systemically healthy (ASA I 2) adult (35male, 25 female; mean age 48.3 8.7 years) patients with bilateral missing first molars of the mandible were recruited. The inclusion criteria included: sufficient bone volume (width 7mm or more, height 10mm or more), with bone density of D2-D3 (Lekholm and Zarb classification) and without active periodontitis and a desire to continue with the recalls. The exclusion criteria were: Uncontrolled diabetes, smoking over 10 cigarettes/day, bruxism, previous bone grafting at the site and immunosuppressive therapy.

##  Randomization and Blinding:

An RN table was created using the computer, which assigned the site of implant design to be right or left molar per patient. Sealed envelopes opened intraoperatively were used to hide the allocation concealment. The statistician and the examining radiologist were still blinded to group assignment. Only one experienced surgeon (15 years of experience, more than 2000 implants performed) performed surgical procedures.

##  Implant systems:

Two titanium implants (4.3 mm diameter, 10 mm long) were commercial pure and were compared:

[1] Tapered Group: Progressive-thread design with thread depth of 1.2 mm apically tapering to 0.6 mm coronally, internal hex connection, SLA surface (Sa 1.8-2 surface)

[2] Straight Group: V-thread design with a depth of 0.8 mm, internal hex connection, the same SLA surface.

## Prosthetic and surgical protocol:

This was done under local anesthesia (4% articaine with 1:100,000 epinephrine) and a vertical release of the alveolar ridge was done in a mid-crestal incision. Sequential osteotomy preparation was in accordance with the manufacturer instructions at 800 rpm under constant irrigation. Torque during the insertion of implant was measured on a calibrated surgical motor with a torque control. Cover screws were applied and non-resorbable sutures were applied to close flaps. Three months later, the second stage surgery revealed implants and healing abutments were installed. Temporary cement was used in standardized zirconia crowns four weeks afterwards.

## Outcome assessments:

Primary stability was assessed right after the insertion by RFA (Osstell ISQ, Göteborg, Sweden) in 2 perpendicular axes (mesio- distal and bucco-lingual), where the means of ISQ values were obtained. The secondary stability was checked at 3, 6 and 12 months after loading. CBCT (Planmeca ProMax 3D, 0.2 mm voxel size) was used to assess marginal bone loss (at baseline (immediately after surgery) and 12 months). A blinded radiologist measured at mesial and distal points between the implant shoulder and the first contact of bone averaged per implant. Implant survival, peri-implant probing depths and insertion torque were the secondary outcomes.

## Statistical analysis:

The analysis was done using SPSS version 25.0. Shapiro-Wilk test was used to check the normality. Paired t-test compared the contralateral implants in terms of ISQ and bone loss. The repeated measures ANOVA were used to evaluate the changes in ISQ with time. Categorical variables were tested using Chi-square test. Pearson correlation compared insertion torque-ISQ relationship. Significance was set at p<0.05. Results are demonstrated in the form of mean + SD or median (IQR).

## Results:

All sixty patients completed the 12-month follow-up, yielding 120 implants with 100% survival. No surgical complications, peri-implant infections, or prosthetic failures occurred. Baseline demographic parameters and implant distribution were balanced (Table 1). Mean bone width was 8.2±0.6 mm and mean height was 11.4±0.9 mm, with no intergroup difference (p=0.892). Primary stability (placement ISQ) was significantly higher for tapered implants (72.4±4.8) versus straight implants (68.9±5.2), with a mean paired difference of 3.5 (95% CI: 2.1-4.9, p<0.001). This advantage persisted at 3 months (75.1±4.2 vs 72.3±4.9, p=0.007). However, ISQ values converged thereafter, showing no significant difference at 6 months (76.8±3.9 vs 75.6±4.4, p=0.112) or 12 months (77.2±3.7 vs 76.5±4.1, p=0.348). Repeated measures ANOVA confirmed a significant time x design interaction (F=8.34, p<0.001), indicating differential stability evolution (Table 2). Twelve-month crestal bone loss was significantly lower for tapered implants (0.82±0.31 mm) compared to straight implants (1.34±0.45 mm), with a mean difference of 0.52 mm (95% CI: 0.38-0.66, p<0.001). Mesial and distal sites exhibited similar patterns (mesial: 0.79±0.29 vs 1.31±0.43 mm; distal: 0.85±0.33 vs 1.37±0.47 mm; both p<0.001). Insertion torque correlated moderately with placement ISQ for tapered (r=0.61, p<0.001) but weakly for straight implants (r=0.34, p=0.008). Peri-implant probing depths at 12 months were comparable (tapered: 2.8±0.6 mm; straight: 2.9±0.7 mm; p=0.523) (Table 3). Subgroup analysis stratified by bone density revealed that in D2 bone (n=42), tapered implants demonstrated 0.58 mm less bone loss (p<0.001), whereas in D3 bone (n=18), the difference was 0.44 mm (p=0.003). No significant gender-related differences were observed (p=0.784).

## Discussion:

This split-mouth randomized controlled trial is suggestive of the fact that tapered macro geometry improves the primary stability and marginal bone loss rate in opposition to straight-walled implants in the posterior mandible. The 3.5 ISQ difference at the placement and 0.52 mm decrease in the crestal bone loss at 12 months have significant clinical implications with regard to implant choice and prognosis. This primary stability of tapered implants is stronger than the predictions of biomechanical stability and previous clinical results [5, 7]. The progressive thread arrangement produces apical compression, augmentation of bone-implant contact, especially in the coronal third which is essential in withstanding micromotion during healing [15]. This mechanical advantage is strongest in the case of placement and they translate to a higher ISQ value. Similar results were observed in a randomized clinical trial comparing two implant systems with differing designs, where tapered implants achieved significantly higher baseline ISQ (72.4 ± 4.8 vs. 68.9 ± 5.2, p<0.001) and reduced 12- month crestal bone loss (0.82 ± 0.31 mm vs. 1.34 ± 0.45 mm, p<0.001), supporting tapered geometry's benefits in posterior mandible under early loading [16]. Nevertheless, the coincidence of the values of ISQ at 6 months shows that secondary stability, which is controlled by the process of osseointegration and not mechanical retention, is design-independent [11]. The significance of maximising macro geometry in the immediate loading situations where preeminence is of primary importance is supported by this biphasic stability pattern, which is, in mechanical early time's biological equivalence in later life. The most clinically relevant finding of the study is the high decrease in marginal bone loss. The difference of 0.52 mm is more than the clinical significance threshold presented by Papaspyridakos *et al.* [17], who proposed the bone loss disparity of over 0.4 mm to affect long-term survival. This strength is probably due to a number of synergistic processes. To begin with, tapered geometry allows more evenly distributed occlusal loads and not only lowers crestal strain foci which induce osteoclastic activity [6]. Secondly, the thread progressive design can ensure a better stress dissipation during functional loading, which maintains the fragile blood flow to the marginal bone [13]. Third, the relative improved initial stability may reduce fibrous encapsulation that is caused by micromotion which leads to bone resorption [4]. Results of comparative literature are inconclusive and this fact gives importance to our rigorous split-mouth design. 0.3mm difference was found in bone loss in favour of tapered implants at 24 months, which is not significant given the lack of power and the inter-individual variability [7]. On the other hand, significant difference was not observed in bone loss in split- mouth study because of the shorter follow-up (6 months) and a different site (premaxilla) [18]. The period of observation (12 months) includes the most vital period of early remodelling and mandibular molar sites are high-load, high- stress conditions in which design variations are more evident [8].

Isolating the macro geometric effects, the topography of surface was the same in groups. This is important as surface changes such as SLP can also affect bone response without regard of shape [12]. These discoveries, therefore, explain why, despite the same micro-roughness, the macro geometry has a substantial role to play in bone preservation. This can have implication on the manufacturing side, indicating that thread design optimization may have more predictable results as compared to seeking out incremental surface modifications on their own. Numerical superiority is not the only form of clinical applicability. The bone loss was reduced to 58 percent (0.52 mm vs 0.52 mm excess of acceptable limits) which may be decisive in the aesthetics or in areas of the body that are vulnerable and bone must be preserved. To compensate a diminished bone engagement, which may provide indications without grafting, the apical compression of tapered implants may be used to place them immediately in extraction sockets [5]. The analysis of D23 bone only is, however, a limitation to our study because it cannot be extrapolated to very dense (D1) or porous (D4) bone, where straight implants may also show similar or better results than our analysis [9]. Restrictions require a wise interpretation. Single operator design increases procedural consistency with reduced generalizability. Though split-mouth controls systemic factors, local anatomical variations between contralateral sites may bring in bias, but is countered with randomization. Although it is the early bone loss that is captured in our 12-month follow, it will not predict long-term behavior, as it requires 3-5 years of data to establish long-term sustainability [9]. Also, the types of implant systems considered do not allow generalizing to all tapered or straight designs, with regard to minute differences in the thread pitch and taper angle, which might produce different results [19]. These results should be confirmed in multi-centers and in various anatomical locations and bone properties in the future. Individualized design selection criteria could be explained using finte element analyses of patient-specific bone density maps. In addition, as it would perfect clinical algorithms, a study on the relationship between macro geometry and loading protocols-immediate and delayed- would be of importance. Bone metabolism genetic combinations may be used to pinpoint subpopulations that will receive optimum benefit with tapered designs [14]. Overall, this test shows that tapered implant macrogeometry does provide quantifiable benefits in primary stability and marginal bone retention over straight-walled designs. These results are consistent with design specific criteria of selection whereby tapered implants in the posterior mandible areas where primary stability and bone conservation is most important are preferred.

## Conclusion:

Under the constraints of this 12-month split-mouth randomized trial, tapered implant design exhibited much higher primary stability at placement and 3 months and much less marginal bone loss (0.52 mm) than a straight-walled implant. The two designs had the same secondary stability and 100% survival. The results support the idea of tapered macrogeometry being a better option in artificial applications of the posterior mandible with better biomechanical features and preservation of bone. The design of implants is an important variable which clinicians ought to take into account during the treatment planning especially in the situations that require maximum initial stability and minimum crestal remodeling.

## Figures and Tables

**Table 1:** Baseline demographic and clinical characteristics

**Parameter**	**Mean ± SD or n (%)**
Age (years)	48.3±8.7
Gender (male/female)	35/25
Bone width (mm)	8.2±0.6
Bone height (mm)	11.4±0.9
Implant distribution	60 tapered, 60 straight
Insertion torque (Ncm)	Tapered: 32.4±5.2, Straight: 28.7±4.9
SD: Standard Deviation

**Table 2:** Implant stability quotient (ISQ) values over time

**Time Point**	**Tapered Implant**	**Straight Implant**	**Mean Difference**	**p-value**
Placement	72.4±4.8	68.9±5.2	3.5	<0.001
3 months	75.1±4.2	72.3±4.9	2.8	0.007
6 months	76.8±3.9	75.6±4.4	1.2	0.112
12 months	77.2±3.7	76.5±4.1	0.7	0.348
Values are Mean±SD;
ISQ: Implant Stability
Quotient (scale 1-100)

**Table 3:** Marginal bone loss and secondary outcomes at 12 months

**Outcome**	**Tapered Implant**	**Straight Implant**	**Mean Difference**	**p-value**
Mesial bone loss (mm)	0.79±0.29	1.31±0.43	0.52	<0.001
Distal bone loss (mm)	0.85±0.33	1.37±0.47	0.52	<0.001
Total bone loss (mm)	0.82±0.31	1.34±0.45	0.52	<0.001
Probing depth (mm)	2.8±0.6	2.9±0.7	0.1	0.523
Implant survival	100% (60/60)	100% (60/60)	-	-
Values are Mean±SD;
mm: millimetres
